# A Distinct Bronchoalveolar Lavage Cytokine Signature Characterizes Nontuberculous Mycobacterial Pulmonary Disease

**DOI:** 10.3390/medsci14040468

**Published:** 2026-08-09

**Authors:** Norio Kodaka, Kayo Watanabe, Chihiro Nakano, Saeko Shinozawa, Takatomo Hirouchi, Yuto Yoshida, Yuka Yamada, Go Yoshizawa, Rubina Morimoto, Hiroto Matsuse

**Affiliations:** Division of Respiratory Medicine, Department of Internal Medicine, Toho University Ohashi Medical Center, 2-22-36 Ohashi, Meguro-ku, Tokyo 153-8515, Japan; norio.kodaka@med.toho-u.ac.jp (N.K.); kayo.sugawa@med.toho-u.ac.jp (K.W.); chihiro.nakano@med.toho-u.ac.jp (C.N.); saeko.shinozawa@med.toho-u.ac.jp (S.S.); takatomo.hirouchi@med.toho-u.ac.jp (T.H.); yuto.yoshida@med.toho-u.ac.jp (Y.Y.); yuka.yamada@med.toho-u.ac.jp (Y.Y.); go.yoshizawa@med.toho-u.ac.jp (G.Y.); rubina.morimoto@med.toho-u.ac.jp (R.M.)

**Keywords:** nontuberculous mycobacteria, IL-8, IL-13, YKL-40

## Abstract

**Background:** Although diagnosis of pulmonary nontuberculous mycobacterial (NTM) disease typically relies on bronchoscopic sampling, microbiologic testing, and histopathologic evaluation, definitive diagnosis is often difficult. This study investigated whether cytokine profiling of bronchoalveolar lavage (BAL) fluid can help differentiate NTM pulmonary disease (NTM-PD) from other diffuse lung disorders. **Methods:** From January 2023 to July 2025, we prospectively evaluated 50 patients presenting with undiagnosed micronodular or diffuse pulmonary opacities. BAL fluid was collected during bronchoscopy and analyzed for cytokines and related biomarkers. Eleven patients were clinically diagnosed with NTM-PD based on American Thoracic Society/Infectious Diseases Society of America criteria. Cytokine and cellular profiles were compared between patients with NTM-PD and those with other diffuse lung disorders. A secondary analysis compared infectious granulomatous disease (NTM-PD; *n* = 11) with noninfectious granulomatous disease (e.g., sarcoidosis; *n* = 8). **Results:** BAL fluid from patients with NTM-PD showed significantly higher levels of interleukin-8 (IL-8), interleukin-13 (IL-13), YKL-40, and neutrophils compared to those with other diffuse lung disorders. In subgroup analysis, IL-8, IL-13, and neutrophil proportions remained significantly elevated in infectious granulomatous NTM-PD compared with noninfectious granulomatous disorders. **Conclusions:** NTM-PD is associated with a distinct BAL cytokine profile characterized by elevated IL-8, IL-13, YKL-40, and neutrophil proportions.

## 1. Introduction

The incidence of nontuberculous mycobacterial pulmonary disease (NTM-PD) has increased substantially worldwide, as demonstrated in multiple population-based studies [1]. Despite this growing burden, diagnosis remains challenging because clinical, radiographic, and microbiologic features often overlap with those of other diffuse lung disorders. In Japan, mortality attributable to NTM-PD has recently surpassed that from pulmonary tuberculosis, underscoring the expanding clinical and public health impact of this disease [2].

Although increased awareness and advances in microbiologic techniques may partly explain the rise in diagnosed cases, persistently poor treatment outcomes likely contribute to the increasing mortality. Current guidelines recommend prolonged multidrug therapy for at least 12 months after culture conversion; however, treatment success rates remain slightly above 50%, and recurrence or reinfection is common [3,4]. Notably, longer treatment durations may reduce mortality, but extended therapy also increases toxicity and treatment burden. These limitations highlight the urgent need for better risk stratification and more individualized therapeutic decision-making.

NTM-PD is a granulomatous lung disease with a heterogeneous natural history. Some patients show minimal radiographic or clinical progression even without treatment [5], whereas others develop progressive lung destruction and may ultimately die [2]. Distinguishing indolent from progressive disease remains a central clinical challenge. Microbiologic confirmation is often difficult, particularly in patients who cannot expectorate sputum or who have mild disease, in whom cultures may remain negative despite active infection. In such cases, bronchoalveolar lavage (BAL) is frequently performed and provides direct access to the affected airway environment. Beyond microbiologic and histopathologic assessment, BAL fluid (BALF) reflects the local immune milieu. Previous studies have demonstrated that mycobacterial infections induce complex airway immune responses involving neutrophils, macrophages, and type 1 cytokines. Additionally, several reports have described elevated IL-13 or related type 2 mediators in granulomatous or chronic inflammatory lung diseases, including mycobacterial infections [6,7,8,9]. Neutrophil-associated cytokines, such as IL-8 and YKL-40, have also been implicated in chronic mycobacterial lung disease and may contribute to tissue remodeling and persistent inflammation. However, few studies have systematically evaluated these cytokines directly in BALF across different pulmonary diseases, and the local airway immune environment in NTM-PD remains poorly characterized.

Herein, we analyzed BALF from patients with micronodular or diffuse radiographic abnormalities and comprehensively quantified cytokines and related immunologic biomarkers. A better understanding of the BAL immune landscape in NTM-PD may provide insight into disease mechanisms and support better diagnostic strategies.

## 2. Materials and Methods

### 2.1. Participants

We prospectively enrolled 50 consecutive patients who presented with undiagnosed micronodular or diffuse pulmonary opacities at our institution from January 2023 to July 2025. All patients underwent bronchoscopy with BAL as part of their diagnostic evaluation. Exclusion criteria were suspected or previously diagnosed lung cancer, a confirmed diagnosis prior to bronchoscopy, inability to undergo bronchoscopy because of hypoxemia, and absence of a planned BAL procedure or informed consent.

In this study, infectious granulomatous disease (IGD) consisted exclusively of NTM-PD, specifically pulmonary *Mycobacterium avium* complex (MAC) infection (10 cases of *M. avium*, 1 case of *Mycobacterium intracellulare*). None of the IGD cases involved tuberculosis or fungal infections. Eleven patients met the American Thoracic Society/Infectious Diseases Society of America diagnostic criteria for NTM-PD. The remaining 39 patients were diagnosed with other diffuse lung disorders, including noninfectious granulomatous diseases (*n* = 8).

### 2.2. BAL Procedure and Cytokine Measurement

BAL was performed using standard techniques in the lung segment showing the greatest radiographic involvement. A portion of each BAL sample was sent to LSI Medience Corporation (Tokyo, Japan) for cytokine and biomarker measurement. The analytes included IL-4, IL-5, IL-6, IL-8, IL-12p70, IL-13, IL-17, IFN-γ, IP-10 (CXCL10), and YKL-40. Differential cell counts were obtained from cytocentrifuged BAL specimens.

Values below the lower limit of quantification were recorded as “below” and excluded from analyses. Values above the upper limit of quantification (ULOQ) were recorded as “above,” and the ULOQ value was substituted for analyses. To avoid treatment-related effects on cytokine measurements, all BAL samples were collected before initiation of antimycobacterial therapy.

### 2.3. Clinical and Laboratory Data

Baseline demographic characteristics and laboratory data were collected at the time of bronchoscopy, including complete blood counts, immunoglobulin levels, C-reactive protein (CRP), lactate dehydrogenase (LDH), and serum protein levels.

### 2.4. Statistical Analysis

Continuous variables are presented as medians with interquartile ranges, and categorical variables as numbers and percentages. Group comparisons were performed using the Mann–Whitney U test for continuous variables and the chi-square test or Fisher’s exact test for categorical variables, as appropriate. We performed an exploratory principal component analysis (PCA) using IL-8, IL-13, YKL-40, and neutrophil counts. The number of components retained was determined using parallel analysis. All analyses were conducted using GraphPad Prism version 9.0 (GraphPad Software, San Diego, CA, USA). Two-sided *p*-values < 0.05 were considered statistically significant.

### 2.5. Ethics Approval and Consent to Participate

All methods were conducted in accordance with the Declaration of Helsinki. The study protocols were approved by the Ethics Committee of Toho University Ohashi Medical Center (approval no. H22003, approved on 18 October 2022,), and written informed consent was obtained from all participants.

## 3. Results

### 3.1. Patient Characteristics

The median age of the cohort was 68.5 years (IQR, 52.8–75.0), and 60% of patients were female. Baseline laboratory values and cytokine profiles are presented in Table 1.

### 3.2. NTM-PD vs. Other Diffuse Lung Disorders

Compared with patients with other diffuse lung disorders, those with NTM-PD had significantly lower peripheral eosinophil counts, lower total white blood cell (WBC) counts, and lower CRP levels.

**Figure 1:**
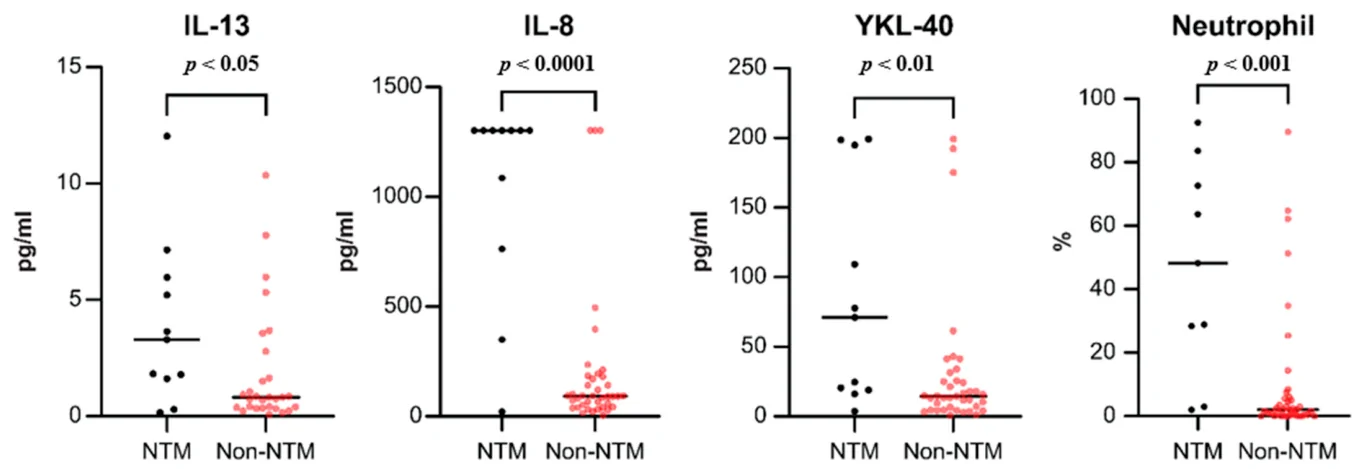
BAL inflammatory markers in NTM-PD vs. non-NTM diffuse pulmonary disorders. Dot plots show individual concentrations of IL-13, IL-8, and YKL-40 (pg/mL) and neutrophil percentages (%) in bronchoalveolar lavage (BAL) fluid from patients with nontuberculous mycobacterial pulmonary disease (NTM-PD) (black dots) and other diffuse lung disorders (non-NTM, red dots). Horizontal lines indicate group medians. Patients with NTM-PD showed significantly higher levels of IL-13 (*p* < 0.05), IL-8 (*p* < 0.0001), YKL-40 (*p* < 0.01), and neutrophil percentages (*p* < 0.001) compared with the non-NTM group.

In BAL fluid, concentrations of IL-8, IL-13, and YKL-40, as well as the percentage of neutrophils, were markedly higher in patients with NTM-PD than in those with other disorders (Figure 1).

### 3.3. NTM-PD vs. Noninfectious Granulomatous Disorders

**Figure 2:**
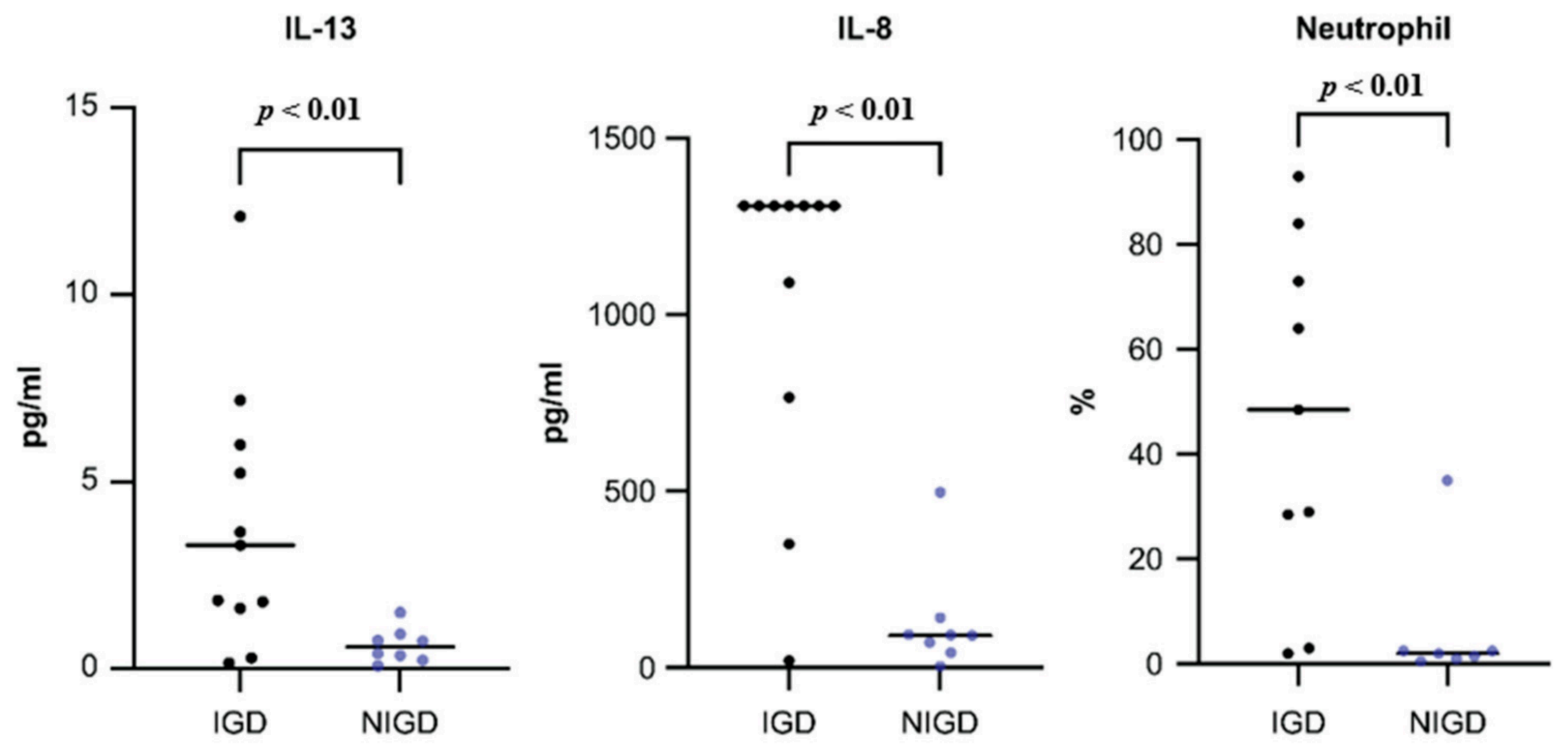
BAL inflammatory markers in NTM-PD vs. noninfectious granulomatous disease. Dot plots show individual concentrations of IL-13, IL-8 (pg/mL) and neutrophil percentages (%) in bronchoalveolar lavage (BAL) fluid from patients with NTM-PD (IGD) (black dots) and noninfectious granulomatous disease (NIGD, blue dots). Horizontal lines indicate group means. IL-13 (*p* < 0.01), IL-8 (*p* < 0.01), and neutrophil percentages (*p* < 0.01) were significantly higher in the NTM-PD group than in the NIGD group.

In the subgroup analysis, BAL concentrations of IL-8 and IL-13 remained significantly higher in patients with NTM-PD than in those with noninfectious granulomatous diseases (NIGD) (Figure 2). Supplementary Tables S1 and S2 summarize detailed comparisons for both analyses.

PCA was performed using IL-8, IL-13, YKL-40, and neutrophil proportions. The number of components to retain was determined using parallel analysis. PC1 explained 76.69% of the total variance, and PC2 explained an additional 14.05%. However, parallel analysis supported retention of PC1 only (Supplementary Figure S1).

## 4. Discussion

In this prospective study of patients with diffuse pulmonary opacities, we observed that NTM-PD is associated with a distinct bronchoalveolar inflammatory signature characterized by elevated levels of IL-8, IL-13, and YKL-40, and increased neutrophil proportions. These findings were consistent when NTM-PD was compared with other diffuse lung disorders and when specifically compared with NIGD. Therefore, NTM infection induces a unique airway immune response that differs fundamentally from that observed in conditions such as sarcoidosis or hypersensitivity pneumonitis [10].

IL-8 is a potent neutrophil chemoattractant, and its elevation in NTM-PD is consistent with the neutrophil-predominant inflammatory response observed in mycobacterial infections, including tuberculosis. In NTM-PD, increased BAL neutrophil proportions have been well documented in prior studies, whereas reports of elevated BAL IL-8 have been limited in the published literature and have largely appeared in conference presentations [10,11,12].

Our findings extend these observations and provide further evidence that NTM-PD represents an IGD characterized by prominent neutrophilic airway inflammation.

YKL-40, a chitinase-3–like protein produced by neutrophils, macrophages, and airway epithelial cells, was also significantly elevated in patients with NTM-PD. YKL-40 is strongly induced by inflammatory cytokines and is associated with chronic inflammation and tissue remodeling [13]. The substantially higher YKL-40 levels observed in NTM-PD compared with NIGD may reflect the chronic and persistent neutrophilic inflammation characteristic of infectious granulomas. Therefore, YKL-40 may serve not only as a biomarker of airway inflammation but also as a potential indicator of ongoing tissue remodeling in NTM-PD.

The elevation of IL-13 in BAL fluid from patients with NTM-PD is particularly noteworthy. IL-13 is increased in many infectious and non-infectious diseases, including mycobacterial infections and granulomatous lung disorders [6,7,8,9]. Our findings extend previous observations by demonstrating that IL-13 is also elevated locally in the airway environment of patients with NTM-PD. Although only one patient in our cohort exhibited cavitary disease, elevated IL-13 levels were observed across the cohort, suggesting that IL-13 may contribute to airway immune responses in NTM-PD beyond cavitary disease. Previous studies have primarily described IL-13 elevation in cavitary tuberculosis; therefore, the presence of increased IL-13 in predominantly non-cavitary NTM-PD suggests that type 2 immune pathways may also be involved in certain contexts of mycobacterial disease. IL-13 in NTM-PD may fit within a broader spectrum of immune responses observed in mycobacterial diseases. Moreover, IL-13 is typically associated with type 2 immune response [14], and its presence in NTM-PD indicates that airway immunity in this condition is more complex than previously recognized. Although IL-13 is not typically linked to mycobacterial immunity, it may contribute to airway remodeling or modulate macrophage function during chronic infection [15]. As IL-13 elevation may also reflect epithelial and mucus-related airway responses that occur independently of cavitary destruction, type-2-associated pathways may be activated even in non-cavitary NTM-PD. Given the small sample size, further studies are needed to validate this finding and to clarify the mechanistic role of IL-13 in NTM-PD.

PCA results suggest that the cytokine and neutrophil profile is largely represented by a single underlying inflammatory pattern. As only PC1 was supported by parallel analysis, interpretation of PC1–PC2 separation was performed cautiously. This multivariate pattern is consistent with the shared inflammatory features observed across IL-8, IL-13, YKL-40, and neutrophil proportions.

Collectively, our findings indicate that NTM-PD is characterized by a predominantly non-type-2 immune response with strong neutrophilic inflammation, accompanied by elevated IL-8 and YKL-40, and unexpectedly increased IL-13. These cytokine patterns may help distinguish NTM-PD from NIGD and may also provide insight into underlying disease mechanisms. A more comprehensive understanding of the cytokine environment in NTM-PD could support the development of targeted immunomodulatory therapies.

The potential therapeutic implications of these findings warrant consideration. In other disease contexts, IL-13 and its receptor, interleukin-13 receptor α2 (IL-13Rα2), have been implicated in malignant tumor progression across various cancers, including melanoma, renal cell carcinoma, adrenocortical carcinoma, and brain tumors [16].

IL-13 blockade, such as with dupilumab, has been associated with reduced respiratory infections in patients with asthma or chronic rhinosinusitis with nasal polyps. Although highly speculative, inhibition of IL-4/IL-13 signaling could theoretically rebalance Th1 responses and thereby enhance host defense against mycobacterial infection. Several reports have described successful use of dupilumab in refractory infections, suggesting that modulation of type-2 immune pathways may have broader antimicrobial effects [17,18]. Furthermore, Onozato et al. reported a case in which treatment of allergic bronchopulmonary mycosis complicated by NTM-PD resulted in improvement of both conditions [19].

Although we observed markedly elevated IL-8 and IL-13 levels in BALF from patients with NTM-PD, the small sample size and single-center design limit the generalizability of our findings. In future studies, it will be important to evaluate temporal changes in cytokines, including whether elevated IL-13 identifies patients with distinct clinical trajectories, such as a tendency toward cavitary progression, and whether IL-13 levels change in response to anti-mycobacterial therapy. These longitudinal assessments may help clarify the clinical significance of IL-13 elevation in NTM-PD. However, this study was not designed to define the specific biological roles of IL-13. Further mechanistic and longitudinal studies are therefore needed to clarify the clinical significance of its elevation.

### Limitations

This study was conducted at a single center with a small sample size, particularly for subgroup analyses. Therefore, the findings require validation in larger cohorts, and future studies should expand this line of investigation to further elucidate the immunopathogenesis of NTM-PD. Although our study identified distinct cytokine and cellular profiles in NTM-PD, we were unable to compare these findings with those from other chronic pulmonary infections. Therefore, we cannot exclude the possibility that some observed features, such as increased neutrophils and elevated IL-8, represent general inflammatory responses associated with chronic mycobacterial infection or IGD rather than a profile specific to NTM-PD. Cytokine measurements were performed using stored BAL samples, and temporal changes in cytokine expression could not be assessed. Owing to the invasive nature of BAL sampling, repeated longitudinal BAL cytokine measurements were not feasible. As this study was exploratory, no formal adjustment for multiple comparisons was applied. Evaluating cytokine trajectories over time, including whether IL-13 levels change with disease progression or in response to anti-mycobacterial therapy, will be an important direction for future research. Larger multicenter studies are needed to validate these findings and to further explore the mechanistic and therapeutic implications of the observed cytokine profiles.

## 5. Conclusions

NTM-PD is associated with a distinct bronchoalveolar inflammatory signature characterized by elevated IL-8, IL-13, YKL-40, and increased neutrophil proportions. These findings provide new insights into the local airway immune environment and may contribute to a better understanding of the immunopathogenesis of NTM-PD. The unexpected elevation of IL-13 represents an important observation that may have clinical implications and warrants further investigation. Although the present study is limited by its small sample size, the findings highlight important immunologic features of NTM-PD. Future studies with larger cohorts will be essential to validate these findings and determine whether IL-13-related pathways may represent potential targets for further therapeutic investigation in NTM-PD.

## Figures and Tables

**Table 1:** Baseline Characteristics of the Study Population (N = 50).

Characteristic	Baseline Value
Age (years)	68.5 (52.8–75)
Female (%)	30 (60)
Never smoking (%)	28 (56)
WBC count	6400 (5300–7900)
Neutrophil percentage	68.7 (61.7–74.2)
Lymphocyte percentage	20.6 (15.25–25.7)
Eosinophil percentage	2.5 (1.25–4.6)
TP (g/dL)	7.5 (6.925–8)
ALB (g/dL)	3.5 (3.05–4.0)
LDH (U/L)	208 (170–267)
CRP (mg/dL)	0.61 (0.17–2.48)
BAL Fluid Findings	
IL-4 (fg/mL)	9.05 (3.47–49.95)
IL-5 (fg/mL)	105.5 (34.5–646.5)
IL-6 (pg/mL)	4.31 (1.26–18.48)
IL-13 (pg/mL)	0.89 (0.39–3.64)
IFN-γ (pg/mL)	1.87 (0.51–7.95)
IL-8 (pg/mL)	98.4 (57.33–564)
IL-12p70 (pg/mL)	0.046 (0.027–0.123)
IP10(CXCL10) (pg/mL)	243 (47.9–1073)
IL17 (fg/mL)	318.5 (102.1–1408)
YKL-40 (ng/mL)	16.29 (7.063–41.35)
BAL neutrophil percentage	3 (0.5–28.5)
BAL lymphocyte percentage	14 (4–30.5)
BAL eosinophil percentage	1 (0–9.5)

Data are expressed as medians (interquartile range) or numbers (%). ALB, serum albumin; BAL, Bronchoalveolar lavage; CRP, serum C-reactive protein; IFN-γ, interferon-gamma; IL, interleukin; IP-10 (CXCL10), interferon gamma-induced protein 10 (C-X-C motif chemokine ligand 10); LDH, lactate dehydrogenase; TP, serum total protein; YKL-40, chitinase-3-like protein 1 (CHI3L1).

## Data Availability

The original contributions presented in this study are included in the article/Supplementary Material. Further inquiries can be directed to the corresponding author.

## Supplementary Materials

The following supporting information can be downloaded at: https://www.mdpi.com/article/10.3390/medsci14040468/s1, Figure S1: Scree plot showing individual and cumulative variance explained by the principal components; Figure S2: Score plot of PC1 and PC2 derived from the principal component (PC) analysis; Table S1: Summary of the principal component (PC) eigenvalues and variance explained; Table S2: Loadings of each variable on the principal components (PC1–PC4).

## Abbreviations

The following abbreviations are used in this manuscript:

ABPM	Allergic bronchopulmonary mycosis
ATS	American Thoracic Society
BAL	Bronchoalveolar lavage
BALF	Bronchoalveolar lavage fluid
CRP	C-reactive protein
CXCL10 (IP-10)	C-X-C motif chemokine ligand 10 (Interferon gamma-induced protein 10)
IDSA	Infectious Diseases Society of America
IFN-γ	Interferon gamma
IGD	Infectious granulomatous disease
IL	Interleukin
IL-4	Interleukin 4
IL-5	Interleukin 5
IL-6	Interleukin 6
IL-8	Interleukin 8
IL-12p70	Interleukin 12 p70 subunit
IL-13	Interleukin 13
IL-17	Interleukin 17
IQR	Interquartile range
LDH	Lactate dehydrogenase
MAC	*Mycobacterium avium* complex
NIGD	Noninfectious granulomatous disease
NTM	Nontuberculous mycobacteria
NTM-PD	Nontuberculous mycobacterial pulmonary disease
Th1	T helper type 1 (cells)
Th2	T helper type 2 (cells)
WBC	White blood cell
YKL-40	Chitinase-3-like protein 1 (CHI3L1)
