# Supplementary material for: A Distinct Bronchoalveolar Lavage Cytokine Signature Characterizes Nontuberculous Mycobacterial Pulmonary Disease
Source: Med Sci (Basel). 2026 Aug 9;14(4):468. doi: 10.3390/medsci14040468 (PMC13515048; doi:10.3390/medsci14040468)

**Supplementary Materials**

**Supplementary Table S1.**

**Summary of the principal component (PC) eigenvalues and variance explained.**

| **PC summary** | **PC1** | **PC2** | **PC3** | **PC4** |
|---|---|---|---|---|
| Eigenvalue | 3.068 | 0.5620 | 0.2984 | 0.07183 |
| Proportion of variance | 76.69% | 14.05% | 7.46% | 1.80% |
| Cumulative proportion of variance | 76.69% | 90.75% | 98.20% | 100.00% |
| Component selection | Selected | | | |

**Supplementary Table S2.**

**Loadings of each variable on the principal components (PC1–PC4).**

| **Variable** | **PC1 loading** | **PC2 loading** | **PC3 loading** | **PC4 loading** |
|---|---|---|---|---|
| IL-8 | 0.9360926 | −0.2547847 | −0.144253 | −0.1949524 |
| IL-13 | 0.882096 | −0.0026982 | 0.4707984 | 0.0157537 |
| YKL-40 | 0.7453561 | 0.6531362 | −0.1336235 | −0.0014938 |
| Neutrophils | 0.9262166 | −0.2655284 | −0.1950495 | 0.1832299 |

**Supplementary Figure S1.**

**Scree plot showing individual and cumulative variance explained by the principal components.**

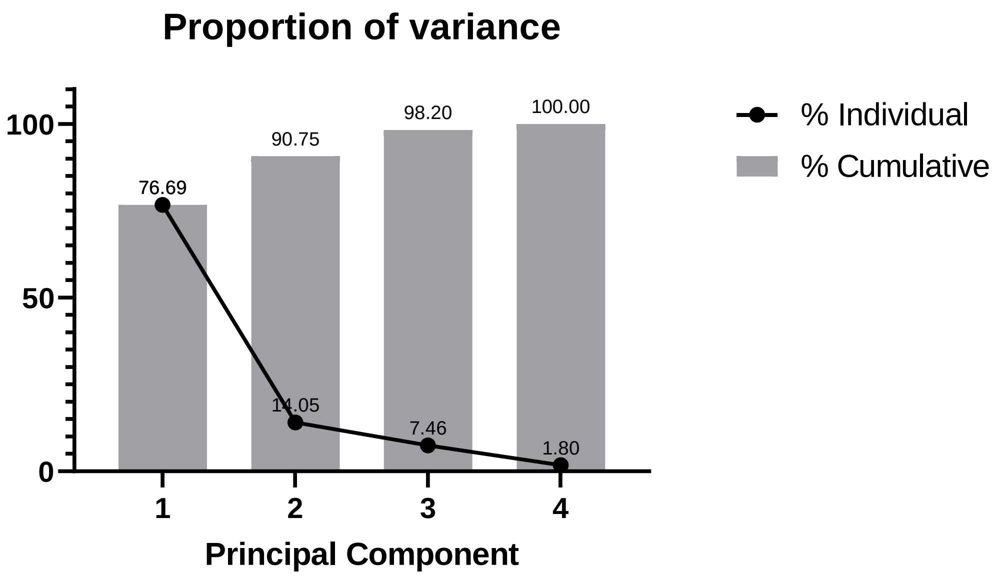

**Supplementary Figure S2.**

**Score plot of PC1 and PC2 derived from the principal component (PC) analysis.**

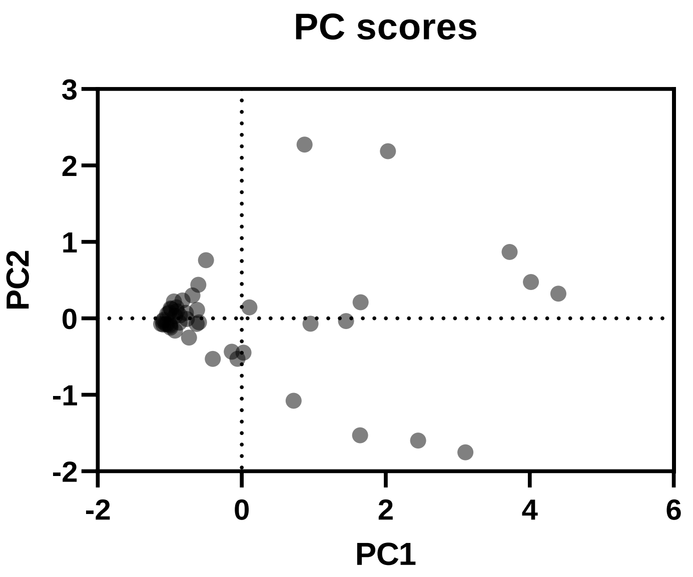

Supplement: Supplementary file 1 [file medsci-14-00468-s001.zip › medsci-4389064-supplementary.pdf]
